# Longitudinal Relationship Between a Novel Alzheimer’s Disease Methylation Index and Cognitive Function

**DOI:** 10.1093/geroni/igaf122.1910

**Published:** 2025-12-31

**Authors:** Nwanyieze Jiakponnah, S Michal Jazwinski, Sangkyu Kim

**Affiliations:** Tulane University School of Medicine / Wake Forest University School of Medicine, New Orleans, Louisiana, United States; Tulane University School of Medicine, New Orleans, Louisiana, United States; Tulane University, New Orleans, Louisiana, United States

## Abstract

Alzheimer’s disease (AD) remains the leading cause of dementia. While existing biomarkers provide insights into AD pathology, they often fall short in early detection and tracking cognitive decline. This study examines the longitudinal relationship between the Alzheimer’s Disease Methylation Index (AD-DMI), a novel, disease-specific epigenetic biomarker, and cognitive trajectories preceding AD diagnosis. Data from 541 ROSMAP participants (mean age = 88 years; 308 AD cases, 233 controls) with up to 16 years of cognitive assessments were analyzed. AD-DMI was developed using dorsolateral prefrontal cortex DNA methylation via elastic-net logistic regression, which selected 100 CpGs that best distinguished AD cases from controls (AUC = 0.79). AD-DMI scores were computed as the average methylation across these CpGs. Multivariable linear mixed-effects models assessed AD-DMI’s association with episodic memory, semantic memory, perceptual speed, visuospatial ability, working memory, and global cognition, adjusting for covariates. Higher AD-DMI scores were significantly associated with steeper decline across all domains (β range: -0.15 to -0.37, q < 0.001). Functional enrichment analysis of AD-DMI CpG-mapped genes identified biological processes related to AD, synaptic function, and autophagy. Eight of these genes overlapped with AD-associated genes previously identified in GWAS. Additionally, AD-DMI scores were significantly associated with increasing methylation at CpGs mapped to APOE, ANK1, and HOXA3 (β range: 0.56 to 2.13, q < 0.01), previously reported as hypermethylated in AD in EWAS. AD-DMI tracks epigenetic signatures linked to AD, supporting its potential for disease monitoring. Future studies should validate AD-DMI in diverse cohorts and assess its non-invasive potential.

